# Ceramic Dressings—A New Non-Pharmacological Therapeutic Option in the Management of Chronic Wounds?

**DOI:** 10.3390/jpm14050498

**Published:** 2024-05-08

**Authors:** Andrzej Hecker, Nikolaus Watzinger, Anna-Lisa Pignet, Birgit Michelitsch, Petra Kotzbeck, Lars-Peter Kamolz

**Affiliations:** 1 Division of Plastic, Aesthetic and Reconstructive Surgery, Department of Surgery, Medical University of Graz, Auenbruggerplatz 29/4, 8036 Graz, Austria; andrzej.hecker@medunigraz.at (A.H.); lars.kamolz@medunigraz.at (L.-P.K.); 2 COREMED—Centre for Regenerative Medicine and Precision Medicine, Joanneum Research Forschungsgesellschaft mbH, Neue Stiftingtalstraße 2, 8010 Graz, Austria

**Keywords:** ceramic dressing, chronic wound, non-pharmacological, bacterial-binding dressings, wound healing

## Abstract

A new ceramic dressing, free from active antimicrobial or pharmaceutical agents, uses physical binding mechanisms for its absorption capacities and bacterial-binding properties. The purpose of this study was to evaluate wound healing, bacterial-related retention, and diagnostic properties of ceramic dressings in patients with stagnated chronic wounds. Methods: In this monocentric, intra-individually controlled, prospective study, patients with conservatively treated refractory chronic wounds were enrolled. One week before the start of the application with ceramic dressing, it was ensured during a screening phase that chronic wounds showed less than a 10% reduction in wound size. During the 4-week ceramic dressing treatment wound size measurements, wound scoring, measurement of wound exudate amount, wound swabs, and ceramic dressing sonication (low-intensity ultrasound) were carried out. The sonication fluid of the removed ceramic dressing was used for analysis of bacterial retention and compared to wound swabs. Results: A total of 20 patients with a mean age of 64.6 years (±26.2) and 21 chronic wounds were included in this study. After a 4-week treatment, a significant reduction of median wound size from 1178 mm^2^ (range 104–6300) to 751.5 mm^2^ (range 16–4819) and better total wound scores were observed (*p* < 0.001). The sensitivity of bacteria detection was 90.7% in the sonication fluid from the ceramic dressings, while only 76.9% in the conventional wound swabs. Conclusion: The new ceramic dressing seems to have a positive impact on wound healing in chronic wounds. Bacteria-binding characteristics of the investigated ceramic dressing, in combination with its debridement, absorption, and detoxification properties, could contribute to its healing abilities. Based on those results, the investigated ceramic dressing seems to be a promising new treatment option for chronic wounds without the use of any active antimicrobial or pharmacological agents. Moreover, ceramic dressings can also be considered for microbiological diagnostic purposes.

## 1. Introduction

Chronic wounds persist as a considerable challenge in clinical practice, significantly affecting patients’ lives and causing a substantial burden on the global healthcare system [1]. Without a “Gold Standard”, current therapies often lead to slow or even failed healing outcomes, which are attributed to the multifactorial pathophysiologic characteristics associated with chronic wounds [2,3]. Many factors may contribute to the delayed healing of chronic wounds. These include, among others, advanced age, nutritional status, presence of underlying chronic diseases, immunocompromised conditions, and bacterial burden [4]. Pathogens such as *Staphylococcus aureus*, *Pseudomonas aeruginosa*, and *β-haemolytic streptococci* are commonly found in chronic wounds [5,6]. These wound pathogens alter the inflammatory response and cause persistent inflammation in the wound area. Consequently, the majority of chronic wounds do not progress beyond the inflammatory stage, resulting in specific delays in healing [6,7,8]. As the degree of bioburden within the wound significantly affects the ability of a chronic wound to heal, regulating bioburden in refractory wound management plays an essential part [8]. One approach to reducing bioburden involves the utilization of systemic and topical antimicrobials. However, in recent years, there has been a growing emphasis on the role of antimicrobial stewardship in wound care to mitigate antimicrobial resistance [5,9]. This approach involves limiting the use of systemic and topical antimicrobials. In response to this, there has been a recent focus on non-medicated materials with bacterial binding properties [10]. Hence, non-medicated dressings with claims of removing microorganisms by irreversibly binding them to their surface to reduce wound bioburden have grown in popularity.

There is increasing evidence supporting the use of bacterial-binding dressings in the treatment of chronic wounds [11]. After binding bacteria, the removal of the dressings can subsequently reduce the number of microorganisms from the wound surface [10]. In contrast to “active” mechanisms, such as damaging the bacterial cell wall through antiseptics and antimicrobials, the physical binding mechanism can help keep the bacterial cell walls of the bound bacteria intact, thereby reducing the release of endotoxins [11]. The presence of these bacterial endotoxins within a wound can cause a prolonged inflammatory phase. As this can lead to the state of a chronic wound, it is all the more important to strive for a reduction of endotoxins in addition to managing resistance developments [12,13]. Nevertheless, a reduction in wound bioburden without the use of an active antimicrobial agent can support the progression of healing in chronic wounds without exacerbating antibiotic resistance problems [11]. A wide range of dressings, including modern dressings with various types of biological activity, is available, but they do not meet the mentioned requirements [14]. The existing literature on non-medicated dressings with assertions of irreversible bacterial binding is mostly in vitro based, and thus, it is limited in scope [10]. Accordingly, more clinical studies are needed to emphasize the benefits of those dressings in terms of bacterial binding properties.

A ceramic wound dressing without any active antimicrobial or pharmacological agents may have these bacteria-binding properties, as postulated by the manufacturer [15]. These ceramic wound dressings are used to absorb and retain large amounts of wound exudate. Strong water-absorbing capabilities have already been demonstrated in this product [16]. This mechanism is supported by the microporous-driven capillary absorption, transport, and storage capabilities of the ceramic dressing [15]. Based on that, ceramic wound dressings can create a moist microenvironment, leading to stimulation of wound healing. In addition to its water-absorbing capabilities, this ceramic dressing also possesses strong endotoxin-binding properties [16]. These detoxification properties, in combination with potential bacteria-binding characteristics, could contribute to its healing abilities in chronic wounds. Nevertheless, the ceramic wound dressing’s healing, bacteria-binding, and retention properties are not yet fully understood. The purpose of this study was to evaluate wound healing, bacterial-related retention, and diagnostic properties of ceramic wound dressings in patients with refractory chronic wounds.

## 2. Materials and Methods

### 2.1. Study Design and Patients

This study was designed as a monocentric, intra-individually controlled, prospective, clinical trial and was conducted at the Division of Plastic, Aesthetic and Reconstructive Surgery, Department of Surgery at the Medical University of Graz between June 2022 and September 2023. The study design and protocol were approved by the institutional ethical review board (Ethical Board Approval No.: 33-275 ex 20/21). This study followed accepted ethical, scientific, and medical standards and was conducted in compliance with recognized international standards, including the principles of the Declaration of Helsinki. Written informed consent was obtained from the participants before study enrolment. The study evaluated wound healing properties (wound size measurement, wound scoring), bacterial-related retention (sonication of ceramic dressing), and diagnostic properties (sensitivity of detected bacteria in wound swab/sonication of ceramic dressing) of ceramic wound dressings in patients with conservatively treated refractory chronic wounds during a 4-week treatment.

Female and male patients aged between 18 and 90 with conventionally treated chronic wounds (at least for 2 months) and stagnated wound sizes were eligible for inclusion in the study. Various types of chronic wounds were considered eligible, including pressure ulcers, diabetic ulcers, Ulcus cruris, and chronic wounds resulting from trauma, infection, or surgical wounds. The definition of wound chronicity used was those wounds that had failed to proceed through physiological phases of wound healing in a timely manner with a minimum duration of eight weeks [8]. To ensure refractory chronic wounds, a one-week screening phase under study conditions, with the application of the standard of care, was conducted before the application started with the ceramic dressing. Within this 1-week screening phase, we defined stagnated wound sizes as a wound size reduction of less than 10 percent. In this screening phase, one week before the application started, wound size reduction was examined, and percentage reduction in wound area was measured after the one-week screening phase. Exclusion criteria were age under 18 years, acute wounds, age of the chronic wound is under 2 months, more than a 10% in chronic wound size within one week after standard of care treatment, systemic infectious effects (e.g., sepsis), allergy to ceramic wound dressings, and skin diseases/dermatoses in the area of the chronic wound (e.g., atopic dermatitis and psoriasis).

### 2.2. Ceramic Wound Dressing

**Figure 1:**
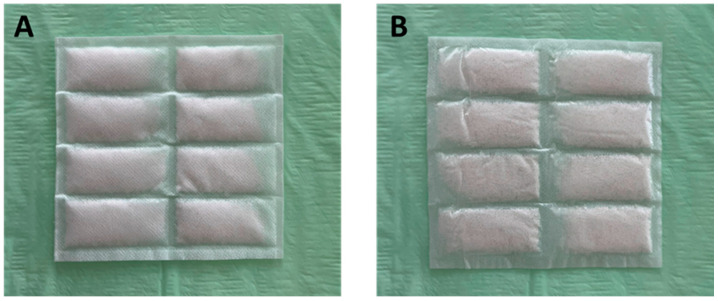
Ceramic dressing (10 cm × 10 cm). (**A**) Non-woven fabric sachet, filled with microporous ceramic granules and sealed in a sterile pouch. This site is outside the wound. (**B**) Shiny and non-sticking side of the sachet is in direct contact with the wound bed.

The ceramic dressings used in this study are commercially available (Cerdak^TM^, Cerdak (PTY) Ltd., Mtunzini, South Africa). They consist of a non-woven fabric sachet, filled with microporous ceramic granules and sealed in a sterile pouch. The shiny and non-sticking side of the sachet is in direct contact with the wound bed (Figure 1). The spherical microporous ceramic granules are loosely packed, allowing free access of air to the wound. Furthermore, this dressing is free from active pharmaceutical ingredients [15,16].

**Figure 2:**
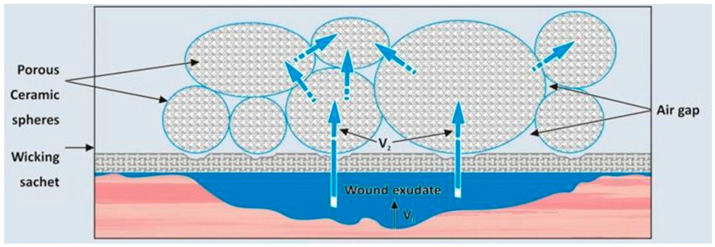
Schematic mechanism of absorption, transport, and storage of exudates by ceramic wound dressing. V_1_ = exudate rate (in kind permission of Cerdak (PTY) Ltd.).

According to the manufacturer, the main feature of the ceramic wound dressing is microporous-driven capillary absorption, transport, and storage of wound exudate. It also involves surface-area-driven adsorption of charged colloids suspended in wound liquids, as well as odorous gases emanating from the wound. The mechanism of absorption, transport, and storage of exudates is schematically illustrated in Figure 2. The wound produces exudate at a rate V_1_. This fluid passes through the wicking sachet and when it comes into contact with the ceramic and its high capillary suction force, it is absorbed at a rate V_2_, which is much faster than the rate of supply (Figure 1). Since each ceramic granule is in contact with surrounding granules with similar high suction potential, moisture migrates continuously between the granules in an attempt to equalize the hydrostatic potential of all the granules in the sachet. There is no driving force for the exudates to leave the ceramic granules, and hence, the interstitial air gaps between the granules remain dry and filled with air [15].

### 2.3. Treatment and Schedule

In eligible patients, the size of included chronic wounds were measured and treated according to the standard of care for one week. After the first week, the wound size was determined again. If there was a wound size reduction of less than 10 percent (stagnated wound size) within one week, patients were included in the study and 4 weeks of treatment with ceramic dressing started. Before application of the ceramic wound dressing, the wounds were cleaned with a wound irrigation solution (Lavasorb^®^, Fresenius Kabi AG, Kriens, Switzerland), and were debrided if clinically necessary. The ceramic wound dressing, big enough to cover the entire wound, was weighted and placed on the wound. The ceramic dressing was placed with the shiny (non-sticking) side of the sachet in direct contact with the wound bed. Dry gauze compresses (Gazin^®^, Lohmann & Rauscher Intl., Rengsdorf, Germany) and adhesive bandages (NOBATEX^®^, NOBAMED Paul Danz AG, Wetter, Germany or Cosmopor^®^ E, Hartmann, Vienna, Austria) were used as secondary dressings and were placed on top to secure the ceramic dressing. In the first three days, ceramic wound dressing change was performed daily. Afterwards, the dressing change was performed every three days (±two days). If clinically necessary, the ceramic wound dressing was changed earlier (e.g., heavily exuding wound). Ceramic dressing weight measurement (before and after application) was performed after every dressing change. Wound size measurement, wound scoring, conventional wound swab, and sonication of ceramic wound dressing were performed before the application started, after 2 and 3 days, and then after 1, 2, 3, and 4 weeks after the initial application of the ceramic wound dressing. For the sensitivity analysis of the two distinct detection methods, pairs of wound swabs and sonication samples (ceramic wound dressing) were examined. A “pair” was defined as a wound swab and sonication taken during the same visit from the same patient.

### 2.4. Study Assessments and Endpoints

#### 2.4.1. Wound Healing Properties and Wound Quality

For wound size measurement, an advanced wound imaging device (eKare inSight^TM^, eKare Europe BV, Nieuw-Vennep, The Netherlands) was used before applying the ceramic wound dressing. Wound scoring was performed with a self-made questionnaire, which included the following wound-related parameters: wound moisture, presence of granulation tissue, pus, crust, erythema, swelling, and necrosis. The total wound scores were calculated as the sum as follows: wound moist (0 = dry, 1 = moist), granulation tissue (0 = wound content full, 1 = wound content half, 2 = wound content empty), pus (0 = not present, 1 = present), crust (0 = fallen off, 1 = wound, 2 = extended, 3 = none), erythema (0 = none, 1, 2, 3, 4 = intense), swelling (0 = none, 1 = medium, 2 = intense), and necrosis (0 = none, 1 = present, 2 = extended 2–3 mm, 3 => 3 mm, 4 = eschar). The wound score ranges from 0 and 17 points, whereby higher wound scores are associated with worse wound quality and impaired healing.

#### 2.4.2. Wound Exudate Weight Measurement

Ceramic wound dressings were weighted with a precision scale before and after application to determine their absorption abilities. Due to the sterile conditions needed for sonication, the second measurement (after removal) was performed on a sterile gauze (Gazin^®^, Lohmann & Rauscher Intl., Rengsdorf, Germany), which will be measured prior adding the ceramic dressing. The weight difference with and without the ceramic dressing yields the absorbed wound exudate weight.

#### 2.4.3. Wound Swabs

Conventional liquid-based wound swabs (ESwab™, Copan Diagnostics Inc., Murrieta, CA, USA) were taken before and after ceramic wound dressing application from the center to the outside of the wound using a zig-zag motion [17].

#### 2.4.4. Sonication

The use of sonication (low-intensity ultrasound) for the disintegration of biofilm on removed implants or dressings and the subsequent culture of the sonication fluid is an alternative method with higher sensitivity compared to conventional microbial cultures for the detection of bacterial strains [18,19]. After dressing removal, dressings with a size of 5 cm × 2.5 cm were placed in a sterile sonicate container (50 mL Falcon^TM^ tube, Fisher Scientific GmbH, Schwerte, Germany) with a 20 mL sterile saline solution (Fresenius Kabi Austria GmbH, Graz, Austria). The closed container was first agitated for 30 s at maximum speed. The container was then placed into the ultrasound bath and was sonicated for one minute at a frequency of 40 kHz and 200 Watt effective power. The container was then again agitated for 30 s to distribute detached biofilm components in the fluid homogeneously. Moreover, 100 μL of sonicate fluid each was transferred to agar plates and incubated at 35 ± 2 °C on McConkey agar (MacConkey II agar [Art. No. 254078; BD, Franklin Lakes, USA]), on blood, CNA, and chocolate agar in humified atmosphere with 5% CO_2_ (Columbia agar + 5% sheep blood [Art. No. 43049, bioMérieux, Marcy-l’Étoile, France], [Columbia CAN agar improved; Art. No. 257306, BD], and Chocolate PolyViteX Agar [Art. No. 43109, bioMérieux]), and under anaerobic conditions on Schaedler and KV agar (Schaedler agar + 5% sheep blood [Art. No. 43401, bioMérieux] and Schaedler-KV Agar [Art. No. 254077, BD]). An additional chocolate agar plate was inoculated with 10 µL of sonicate fluid for quantification of colony counts. A total of 3 mL of sonicate fluid was transferred to a thioglycolate enrichment broth (Thioglycollate with Hemine and Vitamine K1 [Art. No. TV5095D, Thermo Fisher Scientific, Bothell, WA, USA]). Aerobic agar plates were visually inspected by skilled examiners after 24 h and 48 h, and anaerobic plates again after 72 h. Enrichment broths were incubated up to five days and were visually checked for turbidity every day. Identification of bacterial and fungal isolates was performed via MALDI-TOF MS analysis using either the MALDI Biotyper system (Bruker Daltonics, Bremen Germany) or the VITEK MS system (bioMérieux).

### 2.5. Statistical Analysis

Prism 9.5.0 (GraphPad Software, LLC, San Diego, CA, USA) was used for statistical analysis. All numeric endpoints were checked for normality by using a Shapiro–Wilk test. The statistical evaluation included means or medians and standard deviations (SD) or ranges of continuous or ordered variables, and relative frequencies of categorical factors. A Friedman one-way repeated measure analysis of variance (ANOVA) by ranks using the variable “treatment time” (baseline vs. 2 weeks vs. 4 weeks) was performed to analyze wound size and wound scoring. A Dunn’s post hoc comparison procedure was used followed by an ANOVA analysis when significant main effects were present. The sensitivity of each detection method was calculated using cross-tabulation. The main analysis was based on an intention-to-treat basis including all participants who completed at least one dressing change. All statistical tests were two-tailed and differences were considered statistically significant when *p* < 0.05.

## 3. Results

### 3.1. Patients and Wounds

A total of 20 patients (12 male and 8 female) with an average age of 64.6 years (SD ± 26.2) and 21 wounds with a median age of 12 months (range 3–432 months) were included in this study. We had two dropouts in this study. One female patient was unwilling to continue after day 3 (visit 3) and one male patient was excluded during the study course because of non-compliance after day 17 (visit 7). A descriptive overview of the distribution of demographic data, wound types, and wound locations is displayed in Table 1. No adverse event associated with the intervention occurred in the study period.

### 3.2. Wound Healing

During the 4-week treatment period, a significant wound size reduction could be observed (*p* < 0.001). A post hoc test revealed significant improvement in wound size reduction after 2 weeks (*p* = 0.04) and after 4 weeks of treatment (*p* < 0.001) (Table 2).

### 3.3. Wound Scoring

Total wound score significantly improved during the four-week study period (*p* < 0.001). A post-hoc test revealed a significant reduction of total wound score after 4 weeks compared to baseline (*p* = 0.008), but no statistical difference was found after 2 weeks (*p* = 0.25). The number of moist wounds (baseline: 100%, 21/21 wounds) was significantly reduced after 4 weeks (79%, 15/19 wounds, *p* = 0.049). There were no significant differences in granulation tissue, pus, erythema, swelling, or necrosis scores after 4 weeks of treatment compared to baseline (Table 2).

### 3.4. Sensitivity of Wound Swab and Sonication

A total of 109 pairs of wound swabs and sonication (ceramic wound dressing) were analyzed. In total, 334 bacteria were found (wound swab and sonication). Here, 41 different bacterial strains were detected via conventional swabs and 52 different bacterial strains via sonication. The bacterial strains most commonly found here were *Staphylococcus aureus*, *Pseudomonas aeruginosa*, *Proteus mirabilis*, *Enterococcus faecalis*, and *Escherichia Coli*. In total, 257 bacteria were detected via conventional swabs leading to a sensitivity of 76.9%. Moreover, 302 bacteria were detectable via sonication, leading to a sensitivity of 90.7%.

## 4. Discussion

This study investigated the potential of a ceramic dressing, a dressing free from active pharmaceutical ingredients, regarding wound healing, bacterial-related retention, and diagnostic properties in 21 refractory chronic wounds during a 4-week treatment period. After a 4-week application of ceramic dressings in stagnated chronic wounds, significant wound size reduction as well as significantly better wound scores were observed (*p* < 0.001). Moreover, the ceramic dressing showed bacterial retention properties and even demonstrated superior diagnostic properties compared to conventional wound swabs. The majority of chronic wounds do not progress beyond the inflammatory stage, which is characterized by poor perfusion, persistent inflammation, and bacterial burden [20,21,22]. These interactions lead to hard-to-heal wounds characterized by stagnated wound sizes. For proper chronic wound management, standard treatment concepts are based on three main areas: debridement, moisture balance, and infection control through bacterial bioburden balance [21]. In the following section, these three main areas concerning the investigated ceramic dressing will be discussed.

### 4.1. Ceramic-Dressings-Related Mechanical Debridement as a First Step in the Management of Chronic Wounds

Wound bed preparation is an important first step in the management of chronic wounds [23]. This can be achieved through mechanical wound bed debridement, a process of removal of the necrotic tissue. Wound debridement helps to optimize the wound bed through the reduction in bacterial load, as well as debris, and drainage of infections [23,24]. The ceramic wound dressing consists of a non-woven fabric sachet that is filled with spherical microporous ceramic granules. This rigid and spherical microporous structure of the ceramic dressing acts out its function as a mechanical micro-debridement agent [15]. Since the sachet is in direct contact with the wound bed in a loosely adhered manner, this enables the mechanical removal of necrotic tissue by the ceramic dressing itself, thereby potentially supporting the cleansing of the wound bed. The mechanical debridement-related outcome was reflected in a significantly improved overall wound score after the 4-week treatment (*p* < 0.001), which is shown in Table 2. Besides wound cleansing, mechanical debridement is an important and effective initial approach to reducing the wound-related bacterial bioburden [25]. The debridement by the ceramic dressing itself appears to be one of the possible factors in improving wound quality and, hereby, ultimately stimulating the wound healing process in our patient cohort.

### 4.2. Moisture Balance through Absorptive Capabilities and Ceramic-Dressing-Related Monitoring Properties

Similar to debridement, adequate wound exudate management with absorbable dressings is mandatory in chronic wound management [26]. The use of dressings that retain more moisture generally supports faster healing when compared with dressings with less moisture retention capabilities [27]. Maintaining moisture balance, which facilitates cellular growth, migration, and interaction of growth factors, cytokines, and chemokines, supports the healing process [28,29]. As a result, more effective re-vascularization and re-epithelialization of the wounds are mitigated. However, excessive fluid retention at the wound site can lead to poorer healing and maceration of the surrounding tissue [30,31]. This is particularly important as chronic wounds are associated with higher levels of exudate production through the persistent inflammatory environment [32]. Elevated wound exudate production negatively impairs the healing process, as it slows down or even prevents cell proliferation, interferes with growth factors, and contains elevated levels of pro-inflammatory mediators, reactive oxygen species, and proteases [32,33]. This can lead to biochemical changes in the wound exudate composition, creating a hostile wound environment that negatively impacts the healing process in chronic wounds [33]. Through their absorptive capabilities, ceramic wound dressings can create a moist microenvironment, leading to a stimulation of wound healing [15,16]. Possibly due to microporous-driven capillary absorption properties, the ceramic dressing retains the wound exudate within itself. By discarding these saturated ceramic dressings, excess wound exudate can simultaneously be removed from the wound bed. Additionally, the moisture balance is further supported by the monitoring properties of the dressing itself. When oversaturated, the ceramic dressings discolor. When discoloration reaches between 50% and 70% of the surface area of the ceramic dressing itself, a dressing change is indicated [15,34]. Besides the absorptive capabilities, such monitoring properties can additionally support better moisture balance management. This supports timely dressing changes, ensuring optimal moisture balance and facilitating effective chronic wound management. At the beginning of the study, all chronic wounds were clinically classified as moist wounds according to our wound scoring. After 4 weeks of treatment with ceramic wound dressing, we observed a significant decrease in the proportion of moist wounds, with only 79% of all chronic wounds remaining moist. Interestingly, this reduction of moist wounds was observed alongside an unchanged amount of wound exudate within the ceramic dressings (*p* = 0.066) over 4 weeks. The microporous-driven capillary fluid-absorption mechanism, combined with its storage capabilities, along with our clinical results, suggest that the investigated ceramic dressing meets the dressing requirements for maintaining moisture balance in the management of chronic wounds [15,16].

### 4.3. Bacterial-Binding Properties of Ceramic Dressing and Its Potential for Diagnostic Purposes

Bacterial bioburden plays an important role in determining the chronic wounds’ healing capacity [8]. The presence of bacteria and their endotoxins in wounds can cause a prolonged elevation of pro-inflammatory cytokines. Consequently, this can result in a non-healing wound state triggered by the prolonged duration of the inflammatory phase [13]. Chronic wounds contain multiple species of bacteria, including *Enterococcus faecalis*, *Pseudomonas aeruginosa*, and *Staphylococcus aureus*, among others [5,35]. Once established, these bacteria can become persistent and resistant to antimicrobial treatment [36]. This arises due to the capability of certain bacterial strains to form biofilms. Bacterial biofilms, which are complex communities of bacteria encased within a self-produced extracellular matrix, represent a particularly challenging aspect of bacterial bioburden in chronic wound management [5,36]. Topical antimicrobials may fail to achieve bacterial eradication or to prevent recolonization, due to the low penetrance within bacterial biofilms. This aspect represents the main cause of chronic wound infections [36,37]. However, using the ability of microbial species to bind to wound dressings is a novel approach in the management of chronic wounds [11]. The ceramic dressings used in this study were sonicated and subsequently cultured with conventional microbial methods. Here, bacteria retention could be confirmed within the ceramic dressings themselves. Sonication of the ceramic dressings has demonstrated superior sensitivity for bacterial detection than conventional wound swaps (90.7% vs. 76.9%). This aspect not only makes dressings, such as ceramic dressings, potentially suitable for diagnostic purposes, but also demonstrated superior sensitivity compared to conventional wound swabs. Based on these results, sonication of dressings could replace conventional wound swabs in the management of chronic wounds in the future. In addition to the better sensitivity in microbiological diagnostics, this approach could also reduce unnecessary wound management-related waste. Instead of discarding the dressings, they can be utilized for diagnostic purposes. According to our knowledge, this is the first study to investigate bacterial retention in wound dressings using sonication. Sonication has only been used for diagnostic purposes in implant-based surgery [18,38,39]. Thus, more studies are needed to confirm the diagnostic abilities of sonicated wound dressings, even though the results are promising. Besides the potential diagnostic purpose, bacterial binding dressing provides an antimicrobial effect without the use of active pharmaceutical agents, which bear the risk of cytotoxicity and endotoxin release from bacteria [11,12]. Taking that into account, strong endotoxin-binding properties of ceramic dressings yield a reduction of endotoxins and prevent the emergence of novel resistances [16,40]. Thus, the benefit of a bacterial binding dressing such as ceramic dressing is to control the wound bioburden, including endotoxins, without using active antimicrobial substances [11]. However, the exact bacterial and endotoxin binding mechanism is still not clear. The microporous-driven capillary absorptive capabilities of the ceramic dressing may play a role in the bacteria and endotoxin binding mechanism, but this was not investigated in this study. Recent studies have already demonstrated these bacterial-binding benefits in clinical settings by effectively reducing bioburden in infected wounds as well as in preventing postoperative infections [12,41,42]. While bacterial retention properties in the ceramic dressing itself could be demonstrated, no quantification of bacterial wound bioburden was performed in our study. Therefore, no statements can be made regarding the impact of ceramic dressing on chronic wound bioburden. In addition to the ceramic dressings’ potential healing properties, our results indicate ceramic dressings can also be considered for diagnostic purposes. The bacteria-binding characteristics, in combination with detoxification, adsorption, and debridement properties, could contribute to its healing abilities in chronic wounds, making ceramic dressing, a dressing without any active antimicrobial or pharmacological agents, a promising new treatment option for chronic wounds.

### 4.4. Future Perspectives

Even though it has been clinically demonstrated that ceramic dressings seem to have a positive impact on wound healing in stagnated chronic wounds, the underlying pathophysiological processes remain elusive. Without using any active antimicrobial or pharmacological agents, the investigated ceramic dressings seem to fulfill the requirements for standard treatment concepts of adequate chronic wound management. Thus, to understand the exact impact of ceramic dressing in the healing process of chronic wounds, a deeper insight into wound-healing-related parameters is necessary. Future studies should take wound-healing-related parameters into account, such as pH, quantification of bacterial bioburden and endotoxins, and wound exudate. Capturing these parameters in combination with clinical data can help elucidate the complexity of the chronic wound healing process in association with ceramic wound dressing [22,32]. Wound exudate analysis is indispensable for a comprehensive, tailored approach to wound healing, enhancing patient outcomes and recovery. Investigating wound exudate regarding selected parameters (e.g., total protein content, protease activities, cytokines, chemokine, growth factors, and matrix metalloproteinase) can help to elucidate the association between wound healing and ceramic dressing treatment [8,32]. Although we were able to demonstrate wound healing properties of the ceramic dressing in a heterogeneous spectrum of chronic wounds subject to different pathophysiology, the examination of ceramic dressings in homogeneous chronic wounds is inevitable for gaining a differentiated insight into the impact of these dressings on wound healing in chronic wounds [2,3]. Thus, we recommend that future studies investigate ceramic dressings across a homogenous spectrum of wound types in a controlled setting with a longer observation period. Especially in studies related to “hard-to-heal wounds”, a larger patient cohort is required to achieve the necessary statistical power. This enables the detection of potential differences and patterns, as well as the possibility of conducting subgroup analyses more effectively.

## 5. Limitations

The limitations of this study include the diverse spectrum of various wound types, etiologies, and ages, the small patient cohort of 20 patients, and the short observation period of 4 weeks. Furthermore, no control group was used in this study. Since bacterial bioburden was not quantitatively investigated in this study, no statements regarding the impact of ceramic dressing on chronic wound bioburden can be made.

## 6. Conclusions

The new ceramic dressing seems to have a positive impact on wound healing in chronic wounds. Bacteria-binding characteristics of the investigated dressing, in combination with debridement, absorption, and detoxification properties, could contribute to its healing abilities in chronic wounds. Based on that, ceramic dressings seem to be a promising new treatment option for chronic wounds without the requirement of any active antimicrobial or pharmacological agents. The ceramic dressing showed bacterial retention properties in sonication and even demonstrated superior diagnostic capability compared to conventional wound swabs. Besides representing a potential new treatment option for chronic wounds, this dressing could be used for microbiological diagnostics. Moreover, no adverse events associated with the ceramic wound dressing have occurred during the study period, indicating a high safety level for patients.

## Figures and Tables

**Table 1:** Demographic variables and wound characteristics.

Patients (N = 20)	
Age (years), mean (SD)	64.6 (±26.2)
Gender, (M:F)	12:8
Wounds (n = 21)	
Age of wound (months), median (range) Wound type Ulcus cruris, n (%) Surgical, n (%) Decubital, n (%) Diabetic, n (%) Thermal, n (%) Trauma, n (%) Infection, n (%)	12 (3–432) 7 (33.4) 4 (19.0) 4 (19.0) 2 (9.5) 2 (9.5) 1 (4.8) 1 (4.8)
Wound location Feet, n (%) Lower leg, n (%) Trochanter, n (%)	11 (52.4) 5 (23.8) 3 (14.3)
Knee, n (%)	2 (9.5)

SD: standard deviation; M: male patients; F: female patients.

**Table 2:** Wound size and wound score over a 4-week treatment period with ceramic wound dressings in chronic wounds.

Total N = 20 Patients/n = 21 Wounds	Baseline (n = 21)	2 Weeks (n = 20)	4 Weeks (n = 19)	*p*-Value
Wound size (mm^2^), median (range)	1178 (104–6300)	934 (72–5002)	751.5 (16–4819)	<0.001
Wound score, median (range)				
Total wound score (0–17)	5 (2–9)	5 (4–9)	4 (3–8)	<0.001
Moist wounds (0–1), n (%) Pus (0–1), n (%) Crust (0–3) Erythema (0–4) Swelling (0–2) Necrosis (0–4) Granulation tissue (0–2)	21/21 (100) 2/21 (9.5) 3 (1–3) 0 (0–2) 0 (0–1) 0 (0–3) 1 (0–2)	19/20 (95) 1/20 (5) 3 (3) 0 (0–3) 0 (0–1) 0 (0–3) 0 (0–2)	15/19 (79) 0/19 (0) 3 (3) 0 (0–2) 0 (0–1) 0 (0) 0 (0–2)	0.049 0.364 0.039 0.22 0.05 0.22 0.10
Wound exudate weight (mg), mean (SD)	6067.2 (±2876.6)	4117.4 (±4645.8)	3993.9 (±3651.5)	0.066

n, number of wounds; N, number of patients; SD, standard deviation. Wound exudate weight at baseline was calculated as the sum of the weight differences observed during the first three treatment days. Wound exudate weight was defined as the difference in weight of the ceramic dressing before and after its application.

## Data Availability

The datasets generated and/or analyzed during the current study are available from the corresponding author upon reasonable request.
